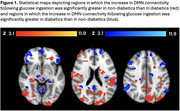# Default mode network connectivity responses to glucose ingestion in cognitively unimpaired older adults with and without type 2 diabetes

**DOI:** 10.1002/alz70856_107508

**Published:** 2026-01-10

**Authors:** Amaryllis A Tsiknia, Victoria R Tennant, Sammantha Nimmo, Choo Phei Wee, Giovanni Trejos, Lorena Contreras, Wendy J Mack, Matthew Borzage, Hussein N Yassine, Meredith N. Braskie

**Affiliations:** ^1^ Mark and Mary Stevens Neuroimaging and Informatics Institute, Keck School of Medicine, University of Southern California, Los Angeles, CA, USA; ^2^ Imaging Genetics Center, Mark and Mary Stevens Neuroimaging and Informatics Institute, Keck School of Medicine, University of Southern California, Marina del Rey, CA, USA; ^3^ Mark and Mary Stevens Neuroimaging and Informatics Institute, Keck School of Medicine, University of Southern California, Marina Del Rey, CA, USA; ^4^ Southern California Clinical and Translational Science Institute, Department of Population and Public Health Sciences, Keck School of Medicine, University of Southern California, Los Angeles, CA, USA; ^5^ Department of Medicine, USC Keck School of Medicine, Los Angeles, CA, USA; ^6^ Keck School of Medicine, University of Southern California, Los Angeles, CA, USA; ^7^ Fetal and Neonatal Institute, Division of Neonatology, Children's Hospital Los Angeles, Department of Pediatrics, Keck School of Medicine, University of Southern California, Los Angeles, CA, USA; ^8^ Center for Personalized Brain Health, Department of Neurology, Keck School of Medicine, University of Southern California, Los Angeles, CA, Los Angeles, CA, USA; ^9^ USC Keck School of Medicine, Los Angeles, CA, USA

## Abstract

**Background:**

Type 2 diabetes mellitus is a risk factor for incident dementia including Alzheimer's disease (AD) dementia, but the mechanism is not clear. Abnormalities in brain default mode network (DMN) connectivity are associated both with T2DM and with increased risk for AD dementia. We explore the response of the DMN to acute glucose ingestion in diabetic versus non‐diabetic older adults to better understand the brain's neurovascular response to glucose and ultimately relate that response to longitudinal cognitive and brain changes.

**Method:**

We scanned (3T Siemens Prisma MRI) *N* = 59 cognitively unimpaired older (50‐65 years) adults (*N* = 26 with T2DM, *N* = 33 non‐diabetic). Each received a fasting resting‐state functional MRI (rs‐fMRI) scan, a 75g glucose tolerance test, a two‐hour break and then a post‐glucose rsfMRI scan. Rs‐fMRI analyses were performed using FSL software with a posterior cingulate cortex/precuneus seed to identify the DMN in each participant. First, we investigated individual participant differences in DMN connectivity between fasting and 120 minutes post‐glucose states to evaluate where each participant had more or less DMN connectivity post‐glucose than while fasting. We then compared those individual differences in the brains’ response to glucose within and between diabetic and non‐diabetic groups. All analyses were adjusted for age and sex. We corrected for voxel‐wise multiple comparisons (cluster‐wise threshold z>3.1; corrected *p*‐threshold = 0.001).

**Result:**

We found significant differences between diabetic and non‐diabetic participants in the brains’ response to glucose ingestion broadly throughout the brain (Figure 1). Within‐group analyses provided context for those differences. In diabetic participants, glucose ingestion caused an increase in DMN connectivity with the cerebellum and superior frontal gyrus but a decrease with parietal, occipital, and orbitofrontal regions. In contrast, in non‐diabetic participants, glucose ingestion caused in increase in DMN connectivity with the lateral occipital cortex and middle temporal gyrus, but a decrease in connectivity with the cerebellum, medial prefrontal cortex, and anterior thalamus.

**Conclusion:**

Our findings highlight distinct DMN connectivity responses to glucose ingestion in older adults with and without T2DM. Relating these differences to longitudinal brain and cognitive changes will help elucidate possible mechanisms and uses of rs‐fMRI connectivity as a predictive biomarker.